# Occurrence of fibrates and their metabolites in source and drinking water in Shanghai and Zhejiang, China

**DOI:** 10.1038/srep45931

**Published:** 2017-04-12

**Authors:** Akiko Ido, Youhei Hiromori, Liping Meng, Haruki Usuda, Hisamitsu Nagase, Min Yang, Jianying Hu, Tsuyoshi Nakanishi

**Affiliations:** 1 Laboratory of Hygienic Chemistry and Molecular Toxicology, Gifu Pharmaceutical University, 1-25-4 Daigaku-nishi, Gifu, Gifu 501-1196, Japan; 2 Faculty of Pharmaceutical Sciences, Suzuka University of Medical Science, 3500-3 Minamitamagaki, Suzuka, Mie 513-8670, Japan; 3 College of Urban and Environmental Sciences, Peking University, No. 5 Yiheyuan Road Haidian District, Beijing, 100871, China; 4 Key Laboratory of Aquatic Science and Technology, Research Center for Eco-Environmental Sciences, Chinese Academy of Sciences, 18 Shuangqing Road, Haidian District, Beijing, 100085, China

## Abstract

Fibrates, which are widely used lipidaemic-modulating drugs, are emerging environmental pollutants. However, fibrate concentrations in the environment have not been thoroughly surveyed. Here, we determined concentrations of the most commonly used fibrates and their metabolites in source water and drinking water samples from ten drinking water treatment plants in Shanghai and Zhejiang, China, using solid-phase extraction and liquid chromatography–tandem mass spectrometry. All the target compounds were detected in at least some of the source water samples, at concentrations ranging from 0.04 ng/L (fenofibrate) to 1.53 ng/L (gemfibrozil). All the compounds except fenofibrate were also detected in at least some of the drinking water samples, at recoveries ranging from 35.5% to 91.7%, suggesting that these compounds are poorly removed by typical drinking water treatment processes. In a peroxisome proliferator-activated receptor α agonistic activity assay, the target compounds showed no significant activity at nanogram per litre concentrations; therefore, our results suggest that the fibrate concentrations in drinking water in Shanghai and Zhejiang, China do not significantly affect human health. However, because of the increasing westernization of the Chinese diet, fibrate use may increase, and thus monitoring fibrate concentrations in aquatic environments and drinking water in China will become increasingly important.

Fibrates are a class of widely used lipidaemic-modulating drugs, the most common being the fibric acids bezafibrate (BF) and gemfibrozil (GF) and the fibrate esters fenofibrate (FF) and clofibrate. In the developed western countries of North America and Europe, many people take these drugs to control hyperlipidaemia resulting from the western diet. Fibrates decrease triacylglycerol concentrations and usually increase high-density lipoprotein concentrations in humans, and these drugs exert their effects by activating peroxisome proliferator-activated receptor (PPAR)α, which is a member of the nuclear hormone receptor superfamily and which plays a pivotal role in controlling fatty acid metabolism and cholesterol homeostasis^1^. All fibrates can be classified as “very persistent”, according to European Union criteria for soil and sediments^2^, and most fibrates are expected to bioaccumulate^3^. Hernando *et al*.^2^ and Sanderson *et al*.^4^ carried out comprehensive quantitative structure–activity relationship screening studies of 2986 pharmaceutical compounds in 51 classes and predicted that fibrates were the most hazardous of the screened compounds. Because fibrates are consumed in high quantities and are environmentally persistent, these drugs and their bioactive metabolites are continuously released into environmental waters, potentially causing adverse effects in aquatic wildlife and humans^5^. However, although many studies have demonstrated the presence of various pharmaceutical and personal care products, including a few fibrates such as BF and GF, in environmental waters^5^,^6^,^7^,^8^,^9^,^10^,^11^,^12^,^13^,^14^, no survey focused on fibrates in wastewater or drinking water has been reported.

Concurrent with recent economic development in China, Chinese dietary habits are becoming more westernized. Consequently, the number of people with lifestyle-related diseases such as hyperlipidaemia and obesity will likely increase, and more people will take fibrates. Thus, pollution of aquatic environments by fibrates and their metabolites will probably worsen in the near future. Additionally, circulation of environmental water in China is poor because the amount of precipitation is low^15^. Thus there is concern that fibrates will accumulate to high concentrations in aquatic environments and that, owing to poor removal efficiency, fibrates will be present in drinking water even after source water has been treated at drinking water treatment plants (DWTPs)^7^. Therefore, it is important to investigate the occurrence of fibrates and their metabolites in aquatic environments and drinking water in China.

For this purpose, we measured the concentrations of BF, GF, and FF and two metabolites [clofibric acid (CA) and fenofibric acid (FA)] (see Supplementary Fig. S1) in source water and drinking water samples collected from ten DWTPs in Shanghai and Zhejiang, China, using a new, highly sensitive method involving ultraperformance liquid chromatography–tandem mass spectrometry (LC-MS/MS) combined with solid-phase extraction (SPE), a method that we developed by modifying a previously reported method^2^,^9^,^16^. In addition, we determined the PPARα agonistic activities of the fibrates and their metabolites by using a cell-based PPARα reporter assay, and we evaluated their bioactivities in the water samples. The results from this study will be helpful in assessing the risks associated with exposure to fibrates in China.

## Results

### Optimisation of ultraperformance LC-MS/MS conditions

The effects of mobile phase composition and additives on the separation of and analytical sensitivity for fibrates and their metabolites were investigated. On the basis of previously reported results^2^, we selected methanol as the organic mobile phase. To improve the retention and separation of fibrates and their metabolites, we used acid-containing ultrapure water as the aqueous mobile phase, and the percentage of the acid (acetic or formic acid) was optimised. Most of the target compounds showed the highest signal intensities with either 0.01% acetic acid or 0.005% formic acid (see Supplementary Fig. S2), and we selected 0.01% acetic acid after considering both separation and sensitivity (see Supplementary Fig. S3a,b). Additionally, we compared the performance of three types of reversed-phase chromatographic columns (C18, C8, and Phenyl columns) in separating fibrates and their metabolites by using methanol and ultrapure water containing 0.01% acetic acid as the mobile phases. The ultraperformance LC-MS/MS selected-reaction monitoring chromatograms of the five target compounds obtained with the three LC columns are shown in Supplementary Fig. S3a,c and d. We selected the C18 column because it provided better separation and peak shapes than the C8 and Phenyl columns, as well as a 1- to 28-fold increase in signal intensity.

Although BF, GF, and CA have been analysed in negative-ionisation (NI) mode using deprotonated [M − H]^−^ molecular ions as precursor ions, we analysed FF and FA in positive-ionisation (PI) mode using protonated [M + H]^+^ molecular ions as precursor ions because the sensitivity has been reported to be higher in PI mode^2^. In contrast, for BF, Stolker *et al*.^8^ found that electrospray ionisation (ESI) in PI mode was more sensitive than ESI in NI mode. Therefore, we compared the suppression of the BF signal in the two ESI modes. In NI mode, signal reductions of 29.1% and 59.8% were observed for extracts of the drinking water and source water samples, respectively; whereas in PI mode, the corresponding signal reductions were lower, 15.8% and 28.2% (see Supplementary Table S1). Therefore, we used the latter mode for BF analysis.

The instrument detection limits (IDLs), the instrument quantification limits (IQLs), the method detection limits (MDLs) and the method quantification limits (MQLs) of all target compounds were shown in Supplementary Table S1.

### Development of SPE method

In a previous study^9^, a weak anion-exchange (WAX), reversed-phase sorbent was used to extract fibrates and their metabolites from wastewater samples, so we evaluated a cartridge with a WAX sorbent in this study. To maximize the extraction efficiency, we compared the performances of four elution solvents: methanol, dichloromethane, acetone, and methanol containing 0.5% NH_4_OH (see Supplementary Fig. S4a). When methanol containing 0.5% NH_4_OH was used, the recoveries of BF, CA, GF, and FA were high (80.4–101%), but the recovery of FF was <40%. When dichloromethane was used, the recovery of FF increased to 65.8%. Therefore, in this study, dichloromethane was used to elute FF from the WAX cartridge, and methanol containing 0.5% NH_4_OH was used to elute the other four compounds.

We compared the performance of the WAX cartridge with the performances of three other types of SPE cartridges—Sep-Pak C18, Oasis HLB, and Oasis MCX—which have previously been used to extract lipid regulators. For these comparisons, the SPE procedure was optimised on the basis of previously described methods^9^,^16^. We found that compared with the WAX cartridge, these three cartridges gave lower recoveries for all the target compounds (see Supplementary Fig. S4b). Therefore, we selected the WAX cartridge for enrichment of the fibrates and their metabolites.

Supplementary Table S1 lists the mean absolute recoveries (*n* = 3) of all the analytes from drinking water and source water samples spiked at concentrations similar to the actual concentrations in the natural environment^7^.

### Fibrate concentrations in source water and drinking water

**Figure 1:**
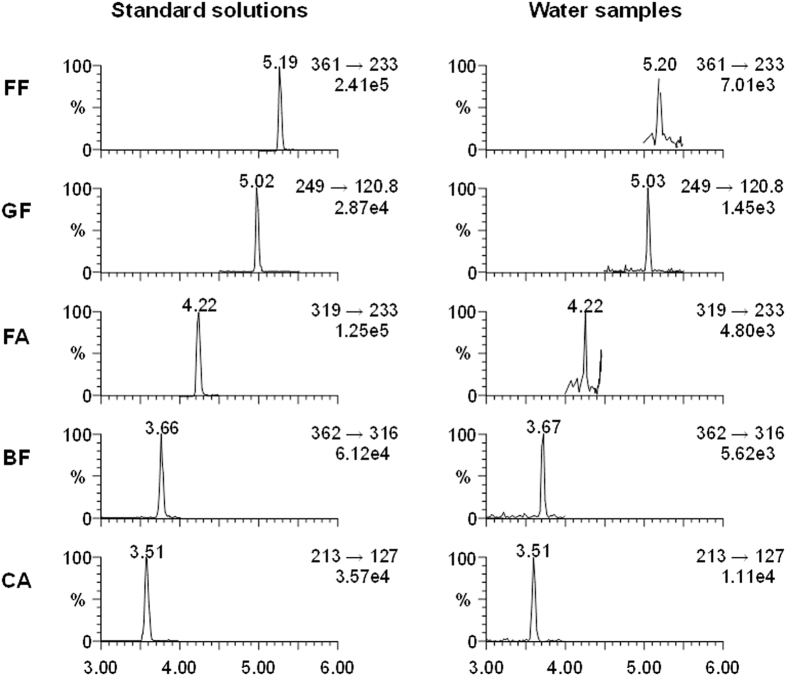
Ultraperformance LC-MS/MS selected-reaction monitoring chromatograms of the five target compounds in standard solutions and water samples. The concentrations in the standard solutions were 1.0 μg/L for bezafibrate (BF), gemfibrozil (GF) and clofibric acid (CA) and 0.1 μg/L for fenofibrate (FF) and fenofibric acid (FA).

The above-described SPE LC-MS/MS method was used to determine the concentrations of the five target compounds in drinking water and source water samples collected from ten DWTPs in Shanghai and Zhejiang, China. Chromatograms of the five analytes in standard solutions and in water samples are shown in Fig. 1, and the analyte concentrations in all the samples are listed in Table 1. All the target compounds were detected in at least some of the source water samples at concentrations ranging from 0.04 to 1.53 ng/L. The most frequently detected compound was CA (in 90% of the samples), followed by GF (80%) and BF (60%). FF and its metabolite FA were detected at lower concentrations than the other fibrates, even though FF is the most widely used of these five fibrates. FF was removed below the detection limit (0.01 ng/L) from source water during water treatment; BF, GF, CA and FA were all present in the drinking water samples at residual ratios ranging from 35.5% to 91.7%.

### Evaluation of bioactivities of fibrates in drinking water

**Figure 2:**
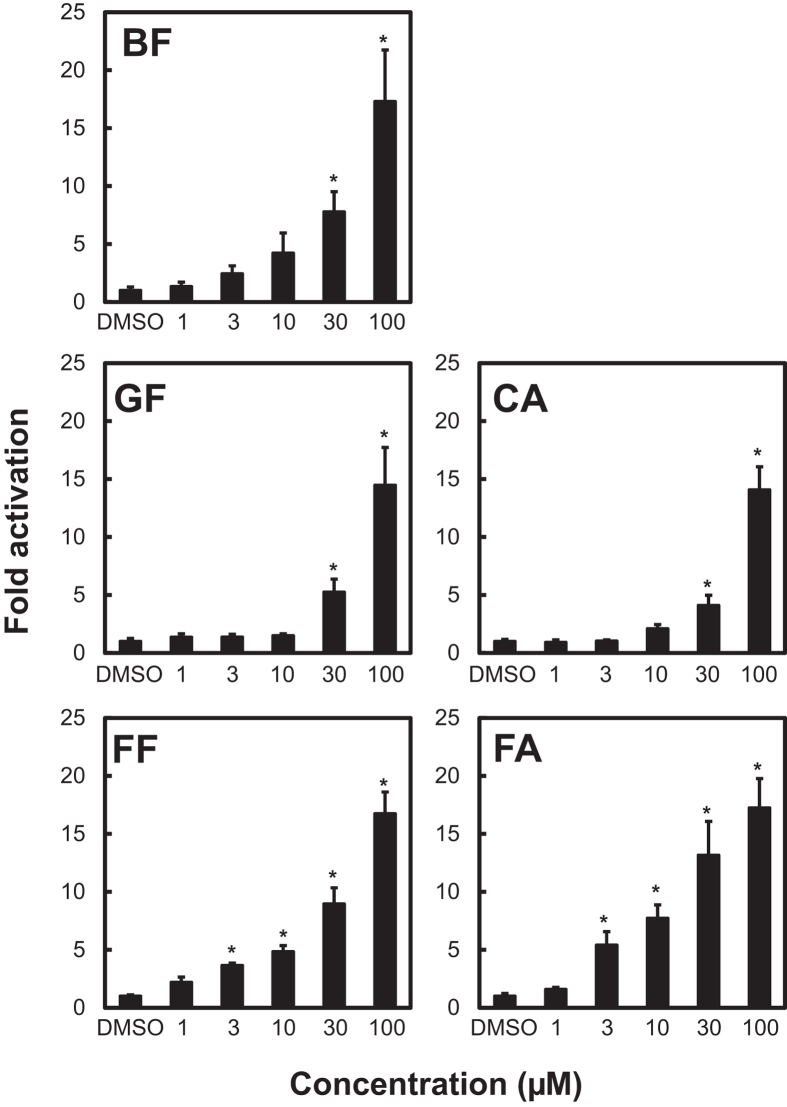
PPARα agonistic activities of the target compounds and their metabolites using a PPARα reporter assay. Hepa 1–6 cells were cotransfected with 20 ng of p4 × UAS-tk-luc and 2 ng of pM-hPPARα full and were then treated with bezafibrate (BF), gemfibrozil (GF), fenofibrate (FF), clofibric acid (CA), or fenofibric acid (FA). pGL-4.74 (0.2 ng) was cotransfected as the internal standard for normalization (see Methods). The results are expressed as average fold activation after normalization to *Renilla* LUC activity. *P < 0.05 vs DMSO, Tukey honest significant difference test.

Because fibrates are PPARα agonists, we evaluated the PPARα agonistic activities of the target fibrates and their metabolites by using a PPARα reporter assay (Fig. 2). All the target compounds showed significant PPARα agonistic activity (*P* < 0.05) and they had different PPARα agonistic activities. The minimum concentrations that showed significant PPARα activity were as follows: 3 μM for FF (equal to 1.08 mg/L, *P* = 0.015), 3 μM for FA (0.96 mg/L, *P* = 0.012), 30 μM for BF (10.9 mg/L, *P* = 0.0027), 30 μM for CA (6.4 mg/L, *P* = 0.0015), and 30 μM for GF (7.5 mg/L, *P* = 0.0056).

To compare our results for China with previously reported fibrate concentrations in drinking (tap) water in Canada and Europe based on the PPARα agonist activity, we calculated the FF equivalent quantity (FF-EQ, ng-FF/L) from the 10% effective concentration (EC_10_) of each compound (see Methods), and we calculated the total FF-EQ by summing the FF-EQ values of the individual compounds (Table 2). The total FF-EQ for drinking water in China was 0.94 ng/L. In Table 2, the country that showed the highest total FF-EQ values was Germany (61 ng/L for tap water and 31 ng/L for drinking water), followed by Canada (9.8 ng/L for tap water), Spain (3.7 ng/L for tap water), and Italy (0.95 ng/L for tap water).

## Discussion

In this study, we used a method involving SPE combined with ultraperformance LC-MS/MS to determine the concentrations of fibrates and their metabolites in source water and drinking water samples collected in Shanghai and Zhejiang, China. The method was highly sensitive, and all the target compounds were detected in at least some of the source water and drinking water samples (Table 1). To our knowledge, ours is the first study focused on the occurrence of fibrates and their metabolites in drinking water in China.

Although a previously reported laboratory-scale experiment showed that chlorination effectively removes pharmaceutical and personal care products from water samples, it was found to be ineffective for removal of fibrates and their metabolites. Specifically, Kubota *et al*.^6^ reported that the concentrations of pharmaceutical and personal care products commonly found in environmental water, such as tetracycline antibiotics (tetracycline, oxytetracycline, chlortetracycline), antipyretic analgesics (diclofenac, mefenamic acid), and fungicides (triclosan), decreased by more than 90% within 4 h of chlorination. In contrast, the removal efficiencies for BF and FF were approximately 50% and that for CA was only 5% under the same treatment conditions. Our results are in good agreement with this previous report. At all ten DWTPs investigated in this study, water was treated by means of chemical precipitation, rapid sand filtration, and chlorination. Our study is the first to indicate that fibrates and their metabolites are not effectively removed from source water during treatment processes typically used at DWTPs in China.

Fibrates act as agonists for PPARα, which serves as a sensor for xenobiotic compounds and exogenous and endogenous lipids to regulate energy consumption, hepatic steatosis, lipoprotein synthesis, inflammation, and development of liver cancer^17^. In fish, some fibrates affect the expression of PPAR-regulated genes involved in lipid homeostasis and induce an embryonic malabsorption syndrome^18^. Furthermore, in humans, sustained exposure to fibrates may increase the risk of hepatocellular carcinoma through a PPARα-dependent or -independent pathway^19^. Because of these adverse effects, the concentrations and bioactivities of fibrates and their metabolites in aquatic environments should be investigated. In this study, we used a reporter gene assay to assess the bioactivity of fibrates in drinking water samples collected in China, and we compared our results with previously reported results for drinking (tap) water samples collected in Canada and Europe (Spain, France, Germany, and Italy) (Table 2). Note that none of the Canadian or European studies focused specifically on fibrates and their metabolites in environmental waters, except for the Canadian drinking water study^10^. The total FF-EQ values calculated from the results of these previous studies were much higher than the total FF-EQ values calculated for China in the current study. For example, for Germany, the total FF-EQ values in drinking water^11^ and tap water^12^ were 31 ng-FF/L (for CA only) and 61 ng-FF/L (for BF and CA), respectively; these FF-EQ values are 2 orders of magnitude higher than the total FF-EQ for the five target compounds in China (0.94 ng-FF/L). In the Canadian drinking water study^10^, only CA was determined, and the FF-EQ (0.20 ng-FF/L) was higher than the FF-EQ range for CA in our study (0.013–0.14 ng-FF/L). Furthermore, the concentrations of the target compounds in source and drinking water in China were at the nanograms per litre level, which is considerably lower than the concentrations required to transactivate PPARα (milligrams per litre). These results suggest that at their current concentrations in China, fibrates have little effect on aquatic wildlife or humans.

However, because of the increase in fibrate consumption that can be expected owing to the increasing westernization of the Chinese diet and because of the poor circulation of environmental water in China due to low precipitation, fibrate pollution in the environment may worsen in China. Additionally, as we have shown, the removal efficiencies of fibrates and their metabolites during typical treatment processes (i.e., chlorination) at DWTPs are quite low. For now, the level of fibrate pollution in drinking water and its source water is lower in Shanghai and Zhejiang than in many developed countries and is of little concern from the standpoint of PPARα agonistic activity. However, considering the factors mentioned above, fibrate concentrations in aquatic environments, in particular source water for and drinking water from DWTPs, should be closely monitored.

## Methods

### Materials

BF, GF, CA, and FF were purchased from Dr. Ehrenstorfer Standards (Augsburg, Germany). From Toronto Research Chemicals (Toronto, ON, Canada), we obtained FA and the following isotopically labelled compounds as internal standards: bezafibrate-*d*_4_ (BF-*d*_4_), gemfibrozil-*d*_6_ (GF-*d*_6_), fenofibrate-*d*_6_ (FF-*d*_6_), clofibric-*d*_4_ acid (CA-*d*_4_), and fenofibric-*d*_6_ acid (FA-*d*_6_).

From Waters (Milford, MA, USA), we purchased an ACQUITY UPLC BEH C18 HPLC column (1.7 μm, 2.1 mm × 100 mm) and the following SPE cartridges: Sep-Pak C18 (6 mL, 1 g, 55–105 μm), Oasis HLB (6 mL, 200 mg, 30 μm), Oasis MCX (6 mL, 150 mg, 30 μm), and Oasis WAX (6 mL, 150 mg, 30 μm). HPLC-grade methanol, acetone, and dichloromethane were obtained from Fisher Chemical (Fair Lawn, NJ, USA). Acetic acid and formic acid (HPLC grade) were purchased from Dima Technology (Richmond Hill, GA, USA). Analytical reagent-grade hydrochloric acid and ammonia solution were obtained from Sinopharm Chemical Reagent Co. (Beijing, China). Ultrapure water was prepared with a Milli-Q Synthesis water purification system (Millipore, Bedford, MA, USA).

All single-standard substances were dissolved in methanol to a concentration of 1000 mg/L and subsequently diluted with methanol to prepare 100 mg/L stock solutions. Then a mixed solution containing the five target compounds was prepared from the stock solutions (10 mg/L for BF, GF, and CA; 1 mg/L for FF and FA). Low-concentration solutions were prepared from the mixed solution by a series of dilutions with methanol. Internal standard solutions were prepared as described for the nonisotopically labelled compounds. All solutions were stored at −20 °C in the dark.

### Cell culture

Mouse hepatoma cells (Hepa 1–6) were obtained from American Type Culture Collection (ATCC CRL-1830; Rockville, MD, USA). The cells were cultured in Dulbecco’s modified Eagle’s medium and 10% foetal calf serum. To determine the effect of fibrates on luciferase reporter gene expression, cells were seeded, precultured for 24 h, and then treated either with fibrates at various concentrations in 0.1% dimethyl sulfoxide (DMSO) or with vehicle alone (0.1% DMSO) for another 24 h. In control experiments, treatment with 0.1% DMSO did not alter luciferase reporter gene expression.

### Plasmid construction

Full-length cDNA of human PPARα was amplified by reverse-transcription polymerase chain reaction using mRNA from HepG2 cells. For the chimeric receptor assay, human PPARα cDNA was fused to the C-terminal end of the GAL4 DNA-binding domain (amino acids 1–147) in the pM expression vector (Clontech, Mountain View, CA, USA) to yield pM-hPPARα. The plasmid constructed was confirmed by sequence analysis. The firefly luciferase (LUC) reporter construct containing four copies of the GAL4 DNA-binding site (upstream activation sequence [UAS]) followed by the thymidine kinase promoter (p4 × UAS-tk-luc) used in the chimeric receptor assay was a gift from Dr Y. Kamei (Kyoto Prefectural University, Japan)^20^.

### Sample collection

Source water and drinking water samples from ten DWTPs in Shanghai and Zhejiang were collected from 4 to 16 January 2012. All samples were collected in 4-L amber glass bottles that had previously been rinsed with methanol and ultrapure water, and the samples were stored at 4 °C during transportation. Five hundred millilitres of each sample was extracted through a glass microfiber filter (GF/C 1.2 μm, Whatman, Maidstone, UK) on the same day.

### SPE method

Water samples (500 mL) spiked with internal standards (1.0 ng/L for BF-*d*_4_, GF-*d*_6_, and CA-*d*_4_; 0.1 ng/L for FF-*d*_6_ and FA-*d*_6_) were passed through the WAX cartridge, which had previously been conditioned with methanol containing 0.5% NH_4_OH (8 mL), methanol (8 mL), and ultrapure water (8 mL). After sample loading, the cartridges were washed with ultrapure water (10 mL), dried under a flow of nitrogen, and then eluted with dichloromethane (8 mL) followed by methanol containing 0.5% NH_4_OH (6 mL). The two fractions were collected in separate amber vials and dried under a gentle nitrogen stream, and the residues were redissolved in methanol (0.5 mL) for ultraperformance LC-MS/MS analysis.

### Ultraperformance LC-MS/MS analysis

The LC apparatus was an ACQUITY UPLC system (Waters). The column was maintained at 40 °C, the flow rate was 0.3 mL/min, and the injection volume was 5 μL. Methanol (solvent A) and ultrapure water containing 0.01% (v/v) acetic acid (solvent B) were used as mobile phases. The gradient was as follows: linear increase from 40% A (initial value) to 100% A within 4 min, maintain at 100% A for 1 min, and then return to 40% A for a 2-min re-equilibration before the next injection. The total run time was 7.0 min.

MS was performed using a Waters Micromass Quattro Premier XE (triple quadrupole) spectrometer equipped with an ESI source (Micromass, Manchester, UK). The optimised MS parameters were as follows: source temperature, 110 °C; desolvation temperature, 350 °C; capillary voltages, −2.5 and + 3.2 kV for ESI in NI and PI modes, respectively; cone voltages, −30 and + 30 V for ESI in NI and PI modes, respectively; desolvation gas flow, 800 L/h; cone gas flow, 50 L/h; multiplier, 650 V. Data were acquired in the selected-reaction monitoring mode, and time-segmented scanning in five functions was used based on the chromatographic separation of the target compounds to maximize the detection sensitivity. The precursor ion, product ions, cone voltage, and collision energies were optimised for each analyte by infusing 1 mg/L standard solutions into the mass spectrometer. The most intense product ion from each precursor ion was chosen as the transition ion for detection and quantitative analysis, and the less intense secondary product ion was used for confirmation^9^. MS/MS conditions for all analytes are summarized in Table 3.

### Quantitation

The five target compounds were identified by comparing the retention times (within ± 2%) and signal-to-noise ratios (within 5) of two selected product ions with those of the corresponding standards. To automatically correct for loss of analytes during sample preparation and for matrix-induced changes in ionisation and to compensate for variations in the instrument response from injection to injection, we used BF-*d*_4_, GF-*d*_6_, CA-*d*_4_, FF-*d*_6_, and FA-*d*_6_ as internal standards. Calibration curves were constructed for the fibrates and their metabolites at the following standard concentrations: 0.05, 0.1, 1.0, 5.0, 10, 25, 50, and 100 μg/L for BF and GF, and 0.01, 0.1, 1.0, 5.0, 10, 25, 50, and 100 μg/L for CA, FA, and FF. The curves were linear with high correlation coefficients (*R*^2^ > 0.99; see Supplementary Table S2).

All equipment was rinsed with methanol and ultrapure water to avoid sample contamination, and laboratory blanks were analysed to assess potential sample contamination.

### Transient transfection assay

Cells were transfected with Lipofectamine (Invitrogen, Carlsbad, CA, USA) in accordance with the supplier’s instructions. Hepa 1–6 cells (3 × 10^4^ cells) were seeded in 24-well plates and precultured at 37 °C for 24 h. The cells were then transfected with pM-hPPARα (2 ng) or p4 × UAS-tk-luc (20 ng). At 24 h after transfection, the five target compounds (0, 1, 3, 10, 30, and 100 μM) were added to the transfected cells, which were then cultured in Dulbecco’s modified Eagle’s medium supplemented with 1% charcoal-stripped foetal calf serum instead of 10% normal foetal calf serum. The cells were harvested 24 h later, and extracts were prepared and assayed for *firefly* LUC activity by using the Dual-Luciferase Reporter Assay System (Promega, Madison, WI, USA) and a Mithras LB940 luminometer (Berthold Technologies, Wildbad, Germany) in accordance with the manufacturer’s instructions. To normalize *firefly* LUC activity for transfection and harvesting efficiency, the *Renilla* LUC control reporter construct pGL 4.74 (Promega) was cotransfected as an internal standard in all reporter experiments. Results are expressed as the average relative *firefly* LUC activity of at least quadruplicate cultures.

### Evaluation of PPARα agonistic activity of fibrates in drinking water

According to the method developed to evaluate the estrogenicity^21^ or retinoic acid receptor α agonistic activity^22^ of environmental samples, the FF equivalent factor (unitless) of each target compound was calculated using the following equation: FF equivalent factor = [FF concentration (ng/L) that gave 10% relative PPARα agonistic activity]/[BF, GF, CA or FA concentration (ng/L) that gave 10% relative PPARα agonistic activity]. The FF-EQ (ng-FF/L) of each sample was determined by multiplying the FF equivalent factor of each fibrate by its concentration (in nanograms per litre) in the sample.

### Statistical analysis

Data were analysed by the Tukey honest significant difference test with SPSS software (version 15.0 for Windows, SPSS Inc., Chicago, IL, USA). Data for DMSO (control) and treatment groups were always obtained from equal numbers of replicate experiments. Values with *P* < 0.05 were considered statistically significant.

## Additional Information

**How to cite this article:** Ido, A. *et al*. Occurrence of fibrates and their metabolites in source and drinking water in Shanghai and Zhejiang, China. *Sci. Rep.* **7**, 45931; doi: 10.1038/srep45931 (2017).

**Publisher's note:** Springer Nature remains neutral with regard to jurisdictional claims in published maps and institutional affiliations.

## Supplementary Material

Supplementary Information

## Figures and Tables

**Table 1:** Concentrations of fibrates and their metabolites in source water and drinking water samples in China.

DWTP^a^	Sample type^b^	Concentration (ng/L) and Residual ratio (%)^c^				
		BF^e^	GF^f^	FF^g^	CA^h^	FA^i^
A	SW	nd^d^	0.18	nd	0.42	nd
	DW	nd	0.08	nd	0.35	nd
	**RR**	**—**	**47**.**4**	**—**	**83**.**5**	**—**
B	SW	nd	0.16	0.05	0.21	nd
	DW	nd	0.07	nd	0.07	nd
	**RR**	**—**	**42**.**8**	**0**	**35**.**5**	**—**
C	SW	0.61	0.13	0.06	0.47	nd
	DW	0.32	0.11	nd	0.34	nd
	**RR**	**52**.**5**	**84**.**2**	**0**	**71**.**5**	**—**
D	SW	0.40	0.18	0.04	0.44	nd
	DW	0.37	0.08	nd	0.34	nd
	**RR**	**91**.**7**	**42**.**5**	**0**	**71**.**5**	**—**
E	SW	nd	nd	0.08	nd	nd
	DW	nd	nd	nd	nd	nd
	**RR**	**—**	**—**	**0**	**—**	**—**
F	SW	0.86	1.53	0.05	0.90	0.32
	DW	0.62	0.56	nd	0.80	0.25
	**RR**	**72**.**2**	**36**.**8**	**0**	**89**.**2**	**78**.**1**
G	SW	0.43	0.07	nd	0.31	nd
	DW	0.30	0.06	nd	0.24	nd
	**RR**	**70**.**6**	**86**.**8**	**—**	**75**.**1**	**—**
H	SW	0.68	0.29	0.07	0.66	nd
	DW	0.56	0.14	nd	0.42	nd
	**RR**	**82**.**4**	**48**.**3**	**0**	**63**.**1**	**—**
I	SW	0.58	0.49	nd	0.49	nd
	DW	0.43	0.26	nd	0.36	nd
	**RR**	**73**.**0**	**52**.**5**	**—**	**72**.**8**	**—**
J	SW	nd	nd	nd	0.37	nd
	DW	nd	nd	nd	0.21	nd
	**RR**	**—**	**—**	**—**	**56**.**8**	**—**

^a^DWTP, drinking water treatment plant; ^b^SW, source water; DW, drinking water; ^c^RR, residual ratio in drinking water after water treatment; ^d^nd, not detected (less than the detection limit); ^e^BF, bezafibrate; ^f^GF, gemfibrozil; ^g^FF, fenofibrate; ^h^CA, clofibric acid; ^i^FA, fenofibric acid.

**Table 2:** Comparison of previously reported fibrate concentrations in drinking (tap) water in Europe and Canada and concentrations for China in the present study.

Country	Sample type (year)	Fibrate^a^	Concentration (ng/L)^b^	FF-EQ (ng-FF/L)^c^	Total FF-EQ (ng/L)^d^	Reference
Spain	Tap water (2012)	BF	nd	nc	3.7	13
		GF	2.0	0.28		
		CA	19	3.4		
France	Drinking water (2007–2008)	BF	0.3–2.2	0.14–0.99	2.7	14
		FA	0.2–1.0	0.35–1.74		
Canada	Drinking water (2003–2004)	CA	0.9–1.1	0.16–0.20	0.20	10
	Tap water (not specified)	GF	70	9.8	9.8	12
Germany	Drinking water (1994–2000)	CA	1–170	0.18–31	31	11
	Tap water (not specified)	BF	27	12	61	12
		CA	50–270	9.0–49		
Italy	Tap water(not specified)	BF	nd	nc	0.95	12
		CA	3.2–5.3	0.58–0.95		
**China**	**Drinking water** (**2012**)	**BF**	**0**.**30–0**.**62**	**0**.**16–0**.**28**	**0**.**94**	**Current study**
		**GF**	**0**.**06–0**.**56**	**0**.**0084–0**.**078**		
		**FF**	**nd**	**nc**		
		**CA**	**0**.**07–0**.**80**	**0**.**013–0**.**14**		
		**FA**	**0**.**25**	**0**.**44**		

^a^BF, bezafibrate; GF, gemfibrozil; FF, fenofibrate; CA, clofibric acid; FA, fenofibric acid; ^b^nd, not detected; ^c^FF-EQ, fenofibrate- equivalent quantity; nc, not calculated; ^d^sum of maximum FF-EQ of each compound.

**Table 3:** Optimized conditions for selected-reaction mode tandem mass spectrometry analysis of fibrates and their metabolites.

Function (min)	Retention time (min)	Compound^a^	Dwell time (s)	Precursor ion (*m*/*z*)	Product ion (*m*/*z*)	Cone voltage (V)	Collision energy (eV)
Negative-ion mode							
3.0–4.0	3.51	CA	0.05	213	126.7^b^	20	14
					84.7		16
4.5–5.5	5.03	GF	0.05	249	120.8^b^	20	12
					126.8		14
Positive-ion mode							
3.0–4.0	3.66	BF	0.05	362	316^b^	30	15
					276		15
4.0–4.5	4.22	FA	0.05	319	233^b^	24	18
					139		30
5.0–5.5	5.19	FF	0.05	361	233^b^	30	16
					139		26

^a^CA, clofibric acid; GF, gemfibrozil; BF, bezafibrate; FA, fenofibric acid; FF, ^b^Product ion used for quantification.
