# Supplementary material for: Occurrence of fibrates and their metabolites in source and drinking water in Shanghai and Zhejiang, China
Source: Sci Rep. 2017 Apr 12;7:45931. doi: 10.1038/srep45931 (PMC5388841; doi:10.1038/srep45931)
Supplement: Supplementary Information [file srep45931-s1.pdf]

Supplementary Information

Occurrence of fibrates and their metabolites in source and drinking water in Shanghai and Zhejiang, China

Akiko Ido[a,1], Youhei Hiromori[a,b,1], Liping Meng[c], Haruki Usuda[a], Hisamitsu Nagase[a], Min Yang[d], Jianying Hu[c,]* and Tsuyoshi Nakanishi[a,]*

[a] Laboratory of Hygienic Chemistry and Molecular Toxicology, Gifu Pharmaceutical University, 1-25-4 Daigaku-nishi, Gifu, Gifu 501-1196, Japan
[b] Faculty of Pharmaceutical Sciences, Suzuka University of Medical Science, 3500-3 Minamitamagaki, Suzuka, Mie 513-8670, Japan
[c] College of Urban and Environmental Sciences, Peking University, No.5 Yiheyuan Road Haidian District, Beijing, 100871, China
[d] Key Laboratory of Aquatic Science and Technology, Research Center for Eco-Environmental Sciences, Chinese Academy of Sciences, 18 Shuangqing Road, Haidian District, Beijing, 100085, China

[1] These authors contributed equally to the research described herein.

* Correspondence and requests for materials should be addressed to T.N. (nakanishi@gifu-pu.ac.jp) or J.H. (hujy@urban.pku.edu.cn).

Supporting Figures

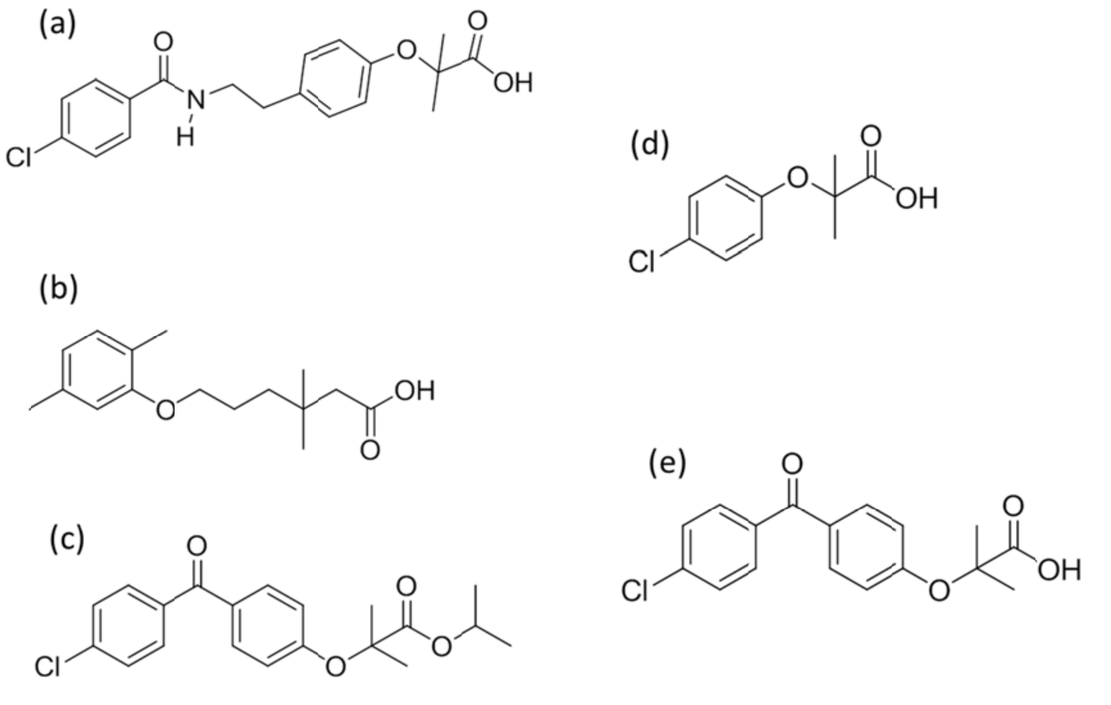

Fig. S1. Chemical structures of fibrates and their metabolites

(a) bezafibrate; (b) gemifibrozil; (c) fenofibrate; (d) clofibric acid; (e) fenofibric acid

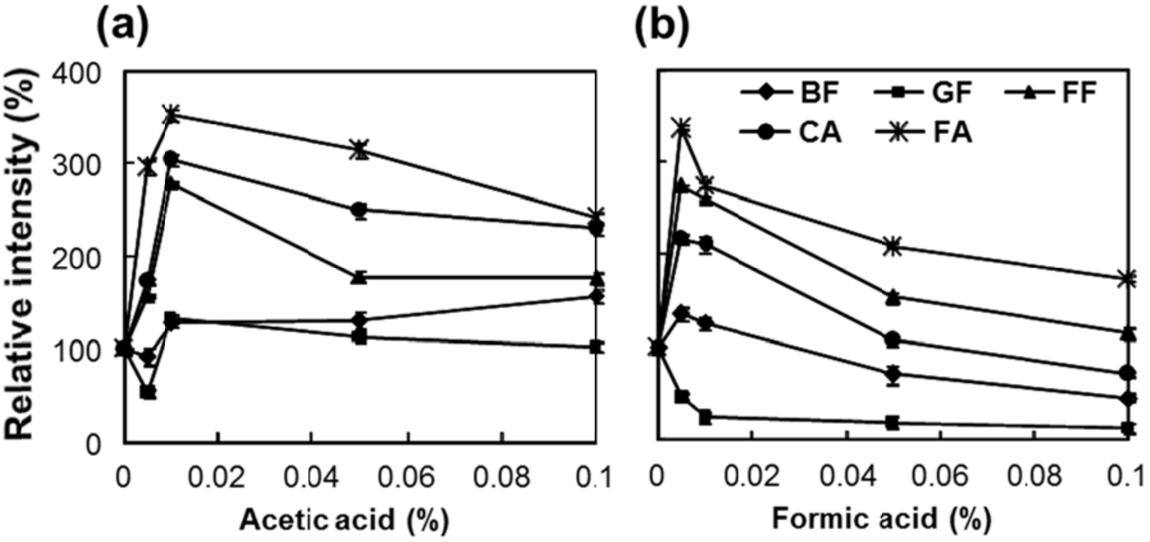

Fig. S2. Effects of percentage of acetic acid (a) and formic acid (b) in the aqueous mobile phase on relative signal intensity

Relative signal intensity is the percentage of the peak area at a given acid percentage (v/v) in the aqueous mobile phase relative to the peak area when ultrapure water was used as the mobile phase. BF, bezafibrate; GF, gemfibrozil; FF, fenofibrate; CA, Clofibric acid; FA, fenofibric acid.

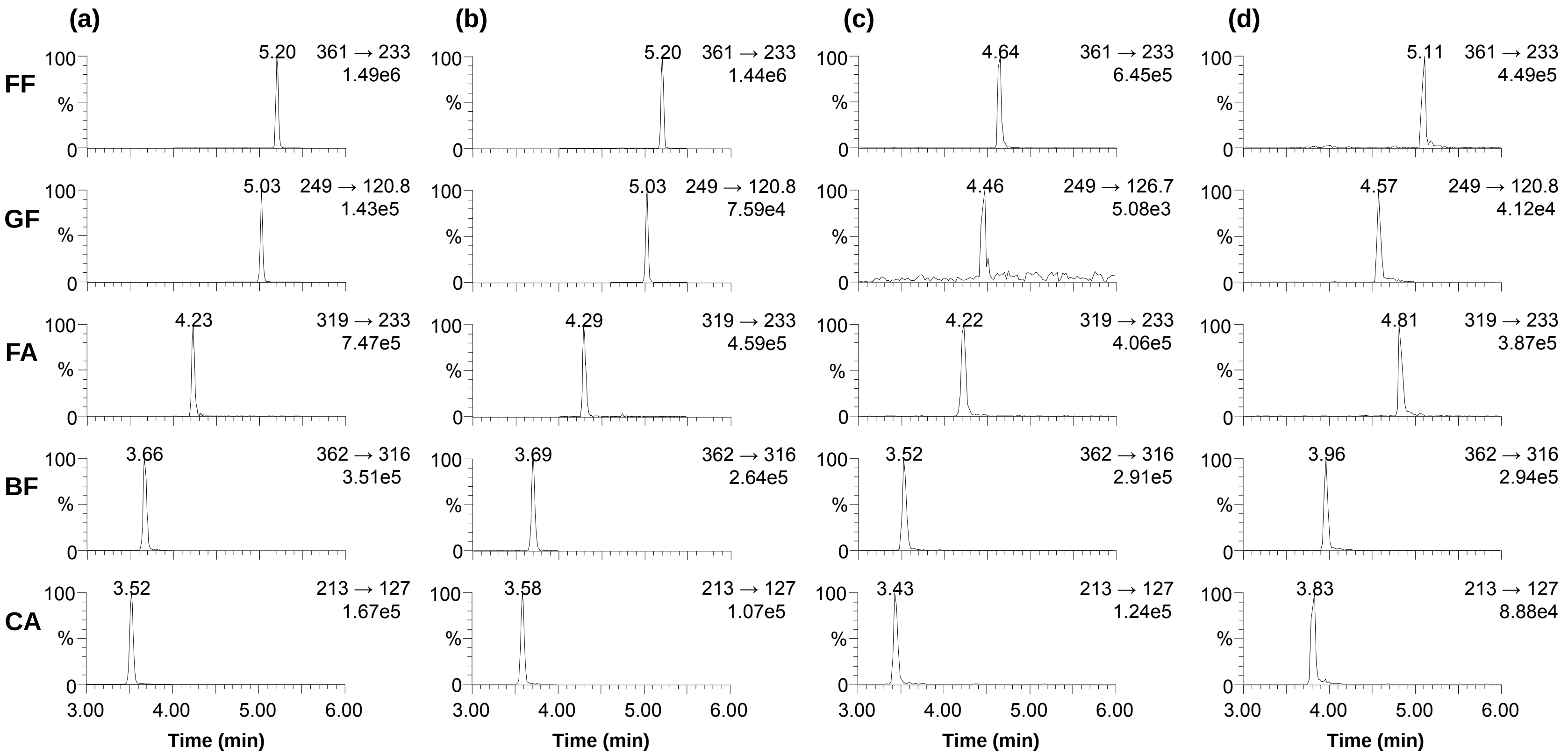

Fig. S3. Liquid chromatography–tandem mass spectrometry selected-reaction monitoring chromatograms of target compounds obtained with various chromatographic columns and mobile phases

(a) Column, C18; mobile phase, methanol/ultrapure water containing 0.01% (v/v) acetic acid. (b) Column, C18; mobile phase, methanol/ultrapure water containing 0.005% formic acid (v/v). (c) Column, C8; mobile phase, methanol/ultrapure water containing 0.01% (v/v) acetic acid. (d) Column, Phenyl; mobile phase, methanol/ultrapure water containing 0.01% (v/v) acetic acid. The concentrations of bezafibrate (BF), gemfibrozil (GF) and Clofibric acid (CA) in the standard solutions were 5.0 μg/L, and those of fenofibrate (FF) and fenofibric acid (FA) were 0.5 μg/L.

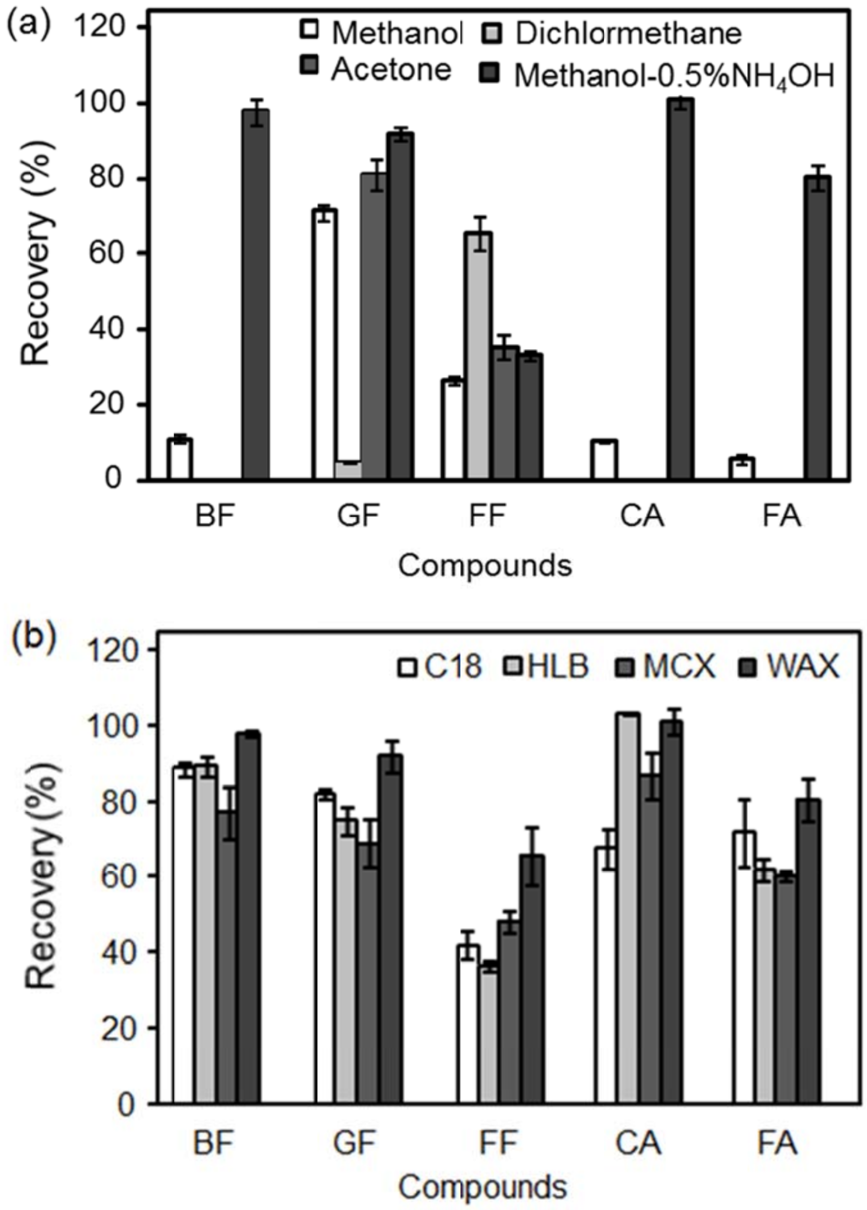

Fig. S4. Recoveries of the five target compounds

(a) a weak anion-exchange (WAX) reversed-phase cartridge with various elution solvents and (b) various solid-phase extraction cartridges [Sep-Pak C18 (C18), Oasis HLB (HLB), and Oasis MCX (MCX)] with MeOH–0.5% NH_4OH and dichloromethane as the elution solvents. BF, bezafibrate; GF, gemfibrozil; FF, fenofibrate; CA, Clofibric acid; FA, fenofibric acid.

Supporting Tables

Table S1. Instrument method sensitivities, recoveries (n = 3), and signal suppression values (n = 3) for fibrates and their metabolites

Compound	Sensitivity (ng/L) [a]						Recovery ± RSD (%) [b]				Signal suppression ± RSD (%) [c]	
	IDL	IQL	MDL		MQL		Absolute recovery		Relative recovery			
			DW	SW	DW	SW	DW	SW	DW	SW	DW	SW
BF [d]	11.2	25.9	0.04	0.07	0.09	0.11	80.4 ± 4.1	71.3 ± 6.1	98.7 ± 2.3	101 ± 3.1	15.8 ± 3.1	28.2 ± 7.5
BF [e]	—	—	—	—	—	—	63.4 ± 3.1	51.0 ± 4.8	—	—	29.1 ± 5.4	59.8 ± 10
GF	14.2	37.1	0.05	0.06	0.08	0.10	74.2 ± 5.6	68.4 ± 12	100 ± 3.6	96.3 ± 1.6	19.8 ± 4.5	32.7 ± 6.5
FF	3.30	17.9	0.01	0.01	0.02	0.03	57.1 ± 8.4	50.8 ± 7.8	102 ± 4.1	99.0 ± 2.1	8.50 ± 1.2	16.0 ± 5.2
CA	8.80	19.2	0.02	0.05	0.05	0.09	83.0 ± 3.2	74.6 ± 5.4	99.5 ± 1.2	98.6 ± 1.4	10.0 ± 2.8	25.8 ± 4.6
FA	7.10	25.2	0.02	0.04	0.05	0.11	71.8 ± 10	60.7 ± 7.5	86.1 ± 6.2	80.3 ± 10	17.2 ± 5.6	31.4 ± 3.1

[a] Recoveries of target compounds were analysed by spiking the source water (SW) and drinking water (DW) samples with a standard solution and the internal standards (n = 3). Instrument detection limits (IDLs) and instrument quantitation limits (IQLs) were determined by means of a signal-to-noise (S/N) approach; standard dilutions reaching ratios of 3 and 10 were used to establish the IDLs and IQLs, respectively. The method detection limits (MDLs) and method quantitation limits (MQLs) were calculated based on the peak-to-peak noise obtained by analysing uncontaminated field samples spiked with different amounts of standards, for minimal S/N values of 3 and 10, respectively. [b] The spike levels were 1.0 ng/L for bezafibrate (BF), gemfibrozil (GF) and Clofibric acid (CA), and 0.1 ng/L for fenofibrate (FF) and fenofibric acid (FA). RSD, relative standard deviation. [c] The signal suppression value for each analyte was calculated using the percentage of signal intensity for a given concentration of analyte in a sample matrix versus that in methanol. The spike levels were 1.0 μg/L for BF, GF and CA and 0.1 μg/L for FF and FA; the corresponding concentrations in real samples were approximately 1.0 ng/L for BF, GF and CA and 0.1 ng/L for FF and FA. RSD, relative standard deviation. [d] BF was analysed in positive-ion mode. [e] BF was analysed in negative-ion mode.

Table S2. Instrumental and method precision values (relative standard deviations, %) and correlation coefficients for calibration curves (R^2)

Compound	Spiked sample concentration (ng/L)	Instrumental precision (%) [a]		Method precision (%) [a]	Correlation coefficient (R^2) [c]
		Intraday (n = 5)	Interday [b] (n = 15)	Intraday (n = 5)	
BF	1	3.4	5.2	2.5	0.996
	10	2.5	3.6	4.7	
	100	0.4	0.5	2.4	
GF	1	7.7	11	9.7	0.994
	10	5.9	6.3	5.6	
	100	2.7	3.8	8.4	
FF	0.1	7.8	6.9	3.2	0.992
	1	3.7	10	2.5	
	10	2.9	3.1	2.1	
CA	1	2.3	6.0	0.5	0.999
	10	2.8	2.6	2.8	
	100	3.2	6.6	1.2	
FA	0.1	7.5	11	6.7	0.997
	1	5.8	5.3	2.1	
	10	3.3	4.6	5.4	

[a] The intraday and interday precision values were calculated as relative standard deviations (%) at three concentrations for each compound within the linear ranges. [b] The instrumental interday relative standard deviations were calculated for day-to-day replicate analyses over a 15-day period. [c] Calibration curves were constructed for fibrates and their metabolites at standard concentrations of 0.05, 0.1, 1.0, 5.0, 10, 25, 50, and 100 μg/L for bezafibrate (BF) and gemfibrozil (GF) and 0.01, 0.1, 1.0, 5.0, 10, 25, 50, and 100 μg/L for fenofibrate (FF), Clofibric acid (CA) and fenofibric acid (FA).
